# Well-Known and Novel Serum Biomarkers for Risk Stratification of Patients with Non-ischemic Dilated Cardiomyopathy

**DOI:** 10.3390/ijms22115688

**Published:** 2021-05-26

**Authors:** Larisa Anghel, Radu Sascău, Ioana Mădălina Zota, Cristian Stătescu

**Affiliations:** 1 Internal Medicine Department, ”Grigore T. Popa” University of Medicine and Pharmacy, 700503 Iași, Romania; larisa.anghel@umfiasi.ro (L.A.); madalina.chiorescu@gmail.com (I.M.Z.); cstatescu@gmail.com (C.S.); 2 Cardiology Department, Cardiovascular Diseases Institute “Prof. Dr. George I. M. Georgescu”, 700503 Iași, Romania

**Keywords:** dilated cardiomyopathy, biomarkers, risk stratification, sudden cardiac death, heart failure

## Abstract

Non-ischemic dilated cardiomyopathy encompasses a wide spectrum of myocardial disorders, characterized by left ventricular dilatation with systolic impairment and increased risk of sudden cardiac death. In spite of all the therapeutic progress that has been made in recent years, dilated cardiomyopathy continues to be an important cause of cardiac transplant, being associated with an enormous cost burden for health care systems worldwide. Predicting the prognosis of patients with dilated cardiomyopathy is essential to individualize treatment. Late gadolinium enhancement-cardiac magnetic resonance imaging, microvolt T-wave alternans, and genetic testing have emerged as powerful tools in predicting sudden cardiac death occurrence and maximizing patient’s selection. Despite all these new diagnostic modalities, additional tests to complement or replace current tools are required for better risk stratification. Therefore, biomarkers are an easy and important tool that can help to detect patients at risk of adverse cardiovascular events. Additionally, identifying potential biomarkers involved in dilated cardiomyopathy can provide us important information regarding the diagnostic, prognostic, risk stratification, and response to treatment for these patients. Many potential biomarkers have been studied in patients with dilated cardiomyopathy, but only a few have been adopted in current practice. Therefore, the aim of our review is to provide the clinicians with an update on the well-known and novel biomarkers that can be useful for risk stratification of patients with non-ischemic dilated cardiomyopathy.

## 1. Introduction

Non-ischemic dilated cardiomyopathy (NIDCM) is a common condition defined as left ventricular dilatation with systolic dysfunction, in the absence of abnormal loading conditions, such as hypertension or valvular disease, or of any epicardial coronary artery disease that can explain the systolic impairment [1,2]. Patients with dilated cardiomyopathy may also have right ventricular dilatation and dysfunction, but this is not necessary for the diagnosis. Unfortunately, dilated cardiomyopathy is relatively common, with an estimated prevalence of up to 1 in 250 of the population, being the leading cause of heart transplantation [1,3]. Depending on the etiology, NIDCM can be classified in at least three main types: genetic/familial, inflammatory/post-inflammatory (e.g., myocarditis), and toxic (e.g., alcohol, chemotherapeutic agents) [4]. Patients with NIDCM have an increased risk of life-threatening arrhythmia and heart failure, the stratification of their risk being a real challenge for the clinician. From the multitude of cardiovascular pathologies which carry significant mortality from sudden cardiac death, NIDCM still remains a common condition. In recent years, multiple risk prediction models have been created in order to predict risk of sudden cardiac death (SCD) in patients with NIDCM, but none have made the current international guidelines. The recommendations of current guidelines are to use the implantable cardioverter-defibrillators (ICDs) for primary prevention of sudden cardiac death in patients with NICDM, depressed left ventricular ejection fraction (LVEF) ≤ 35% and symptomatic heart failure [5,6]. However, we consider that these criteria are unfortunately not sufficient for risk stratification of patients with NIDCM, because some patients with a reduced LVEF may not be at a high risk for SCD and are unjustifiably exposed to the ICDs’ untoward effects, while other patients with a LVEF > 35%, who do not fit with an ICD, according to current indications, might suffer an SCD, and thus, remain unprotected [7]. Therefore, risk stratification in patients with NIDCM is a complex decision, and the New York Heart Association (NYHA) classification system seems to be an unreliable predictor of adverse outcomes. The NYHA functional classification serves as a fundamental descriptor of heart failure and determines clinical trial eligibility and candidacy for drugs and devices. Based on how much the patients are limited during physical activity, NYHA functional classification places patients in one of four categories:

- class I: ordinary physical activity does not cause undue fatigue, palpitation or dyspnea.
- class II: ordinary physical activity results in fatigue, palpitation or dyspnea.
- class III: less than ordinary physical activity causes fatigue, palpitation or dyspnea.
- class IV: unable to carry out any physical activity without discomfort and patients have symptoms of heart failure at rest [6].

In the last few years, significant advances have been made in emerging techniques that can provide useful risk prediction, such as genetic, imaging, cardiopulmonary exercise testing variables or circulating biomarkers. The measurement of biomarkers is of great importance for risk stratification and to find new therapeutic strategies to improve the prognosis. Although they are not specific to NIDCM, they can be of great importance, and one possible way to make them clinically useful is to make use of a DCM-related biomarker modeling score system.

Morrow and de Lemos [8] established some criteria for a biomarker to be used clinically:

- The measurement of the biomarker can be repeated with cost-efficient methods.
- It must bring more information compared to other tests already performed.
- It should be clinically useful for decision making.

**Figure 1:**
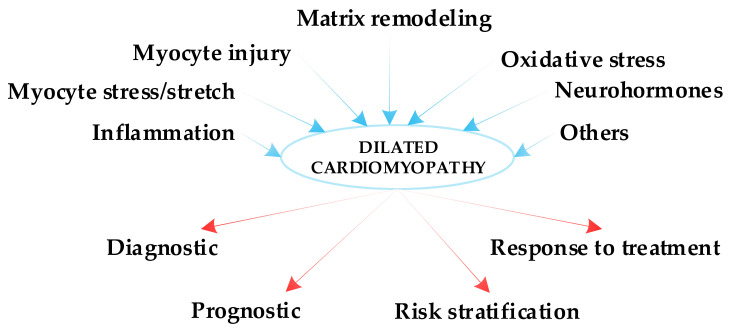
Biomarkers of dilated cardiomyopathy and their implications in clinical practice.

It is quite challenging to categorize the biomarkers that can be used for risk stratification of patients with dilated cardiomyopathy. To date, there is no generally accepted classification of these biomarkers, and the often-used classification in all the studies on this issue is the one mentioned by Braunwald [9]. Another review, published more recently by Dookhun and coworkers, classified these biomarkers based on their pathogenesis [10]. We organized our sections according to the classification mentioned by Braunwald [9] as follows: biomarkers of inflammation, myocyte stress/stretch, myocyte injury, extracellular matrix remodeling, oxidative stress, neurohormones and unclassified biomarkers [9] (Figure 1). All the data presented in this review are our own interpretation of the current data on this issue, and we highlighted the importance and the implications of each biomarker in risk stratification of patients with NIDCM. Additionally, we included more recent data on the usefulness of well-known biomarkers in risk stratification of patients with NIDCM and we have introduced new biomarkers, such as circulating microRNAs, syndecan-1, tumor necrosis factor-α, T-cadherin or growth differentiation factor-15, that have been proven effective in small clinical studies, but which appear to have important benefits for the risk stratification of these patients.

This review describes the latest data on serum biomarkers that are correlated with prognosis as well as data on novel potential biomarkers that can aid in risk stratification of patients with non-ischemic dilated cardiomyopathy.

## 2. Inflammatory Biomarkers

### 2.1. C-Reactive Protein (CRP) and High-Sensitivity-CRP (hs-CRP)

C-reactive protein is a nonspecific biomarker of inflammation and a well-known causative factor of endothelial dysfunction [11]. As observed in other chronic diseases, such as systemic arterial hypertension, diabetes, cancer or chronic kidney disease, the inflammatory process is also observed in heart failure [12,13]. Studies have shown that patients with chronic heart failure (CHF), regardless of the etiology, have elevated serum levels of hs-CRP [14,15,16]. It was also observed that patients with acute heart failure and elevated CRP levels require more frequent hospitalization in the cardiac intensive care unit and, at the same time, have a higher rate of in-hospital mortality [17]. Additionally, considering that CRP is a causative factor of endothelial dysfunction, which also has an important role in the pathogenesis and prognosis of CHF, this inflammatory biomarker can be used as a prognostic indicator of CHF patients with dilated cardiomyopathy [16,18]. There is evidence that hs-CRP is increased in DCM patients in NYHA classes III and IV, with left ventricular systolic dysfunction and more dilated right ventricular and left atrial diameters. Additionally, hs-CRP is an independent predictor of all-cause mortality in DCM patients [19,20]. Another study that evaluated the prognostic role of hs-CRP levels in patients with DCM, as well as their impact on adverse effects during long-term follow-up, was published by Sadahiro and collaborators. They performed a retrospective study which included data from 76 patients with DCM who have been admitted for acute heart failure. After a mean follow-up period of 813 ± 54 days, one of their major findings was that, among patients with DCM and late gadolinium-enhancement (LGE) cardiovascular magnetic resonance, prolonged elevation of hs-CRP at a stable state was the strongest predictor of all-cause mortality and hospitalization [21]. LGE cardiac magnetic resonance is currently the non-invasive gold standard for the identification and quantification of myocardial scar. It is used to assess replacement fibrosis, giving important information regarding the etiology and clinical outcome of patients with DCM [5,6]. The prognostic role of plasma CRP levels specifically in patients with NIDCM was demonstrated in a study published by Chitose and coworkers. Measuring the CRP values in 84 patients with chronic heart failure, after a mean follow-up period of 42 months, they observed that NIDCM patients with high plasma CRP values have a poor prognosis, independent of other parameters [16]. All these results suggest that the CRP value is a useful prognosis biomarker of DCM patients, and there are few data regarding the utility of plasma CRP levels, as a prognostic marker, specifically in patients with NIDCM.

### 2.2. Neutrophil/Lymphocyte Ratio (NLR)

It is known that inflammation has an important role in the pathophysiology and progression of heart failure, its presence indicating a poorer prognosis [13]. Additionally, patients with heart failure and elevated white blood cell count, neutrophilia and lymphocytopenia, have an increased mortality [22,23]. The neutrophil to lymphocyte ratio has recently emerged as a new inflammatory biomarker that can be used in the staging prognosis of several chronic diseases [12,24]. Thus, in the last few years, NLR has been shown to be an eligible biomarker associated with the severity of chronic heart failure in children, adolescents [12] and adults with dilated cardiomyopathy [22]. Avci and coworkers prospectively evaluated 87 patients with idiopathic dilated cardiomyopathy, in order to assess whether NLR levels are associated with echocardiographic parameters, New York Heart Association functional class or B-type natriuretic peptide (BNP) values. They observed a significant association between NLR values and impaired echocardiographic indexes and functional capacity. Additionally, NLR is a significant and independent predictor of increased BNP values, so it may be a useful biomarker of severe clinical evolution in patients with idiopathic DCM [22]. Another recent study evaluated the correlation between NLR and BNP levels and chronic heart failure severity, in children and adolescents with dilated cardiomyopathy. They included 57 patients, with an average age of 48 months, and after a follow-up period of 64 months, concluded that NLR levels are associated with a poor prognosis and a higher risk of death or cardiac transplant [12]. The results of these studies, even though they are few and with a relatively small number of patients, demonstrated that NLR is a noninvasive and available biomarker that may be used to assess the severity of heart failure in patients with dilated cardiomyopathy, but unfortunately, its importance has not been studied specifically in patients with NIDCM.

### 2.3. Galectin-3 (Gal-3)

Galectin-3 is an inflammatory biomarker of which the values are elevated in patients with myocardial inflammation and fibrosis [25]. This protein is released as a consequence of tissue stress in many organs, including the heart, and causes myofibroblast activation [1]. This results in excessive collagen in hypertrophied heart and cardiac dysfunction. It is known that myocardial fibrosis is common in patients with dilated cardiomyopathy. Its presence suggests more severe heart disease and is associated with a more reserved prognosis, due to accelerated heart dysfunction [26]. Additionally, myocardial fibrosis plays an important role in the genesis of ventricular arrhythmia, through reentry mechanisms, increasing the risk of sustained ventricular tachycardia or ventricular fibrillation [27]. The relationship between galectin-3 values and myocardial fibrosis in patients with nonischemic dilated cardiomyopathy was first described by Vergano and collaborators. They evaluated 150 patients with NIDCM and observed that galectin-3 is associated with late gadolinium enhancements at cardiac magnetic resonance imaging. This suggests that galectin-3 is involved in cardiac fibrosis and remodeling in patients with NIDCM, and can, thus, be used in the selection of high-risk patients [25]. In another study, Hu and coworkers aimed to investigate whether LGE associated with galectin-3 values offer more precise prognosis for patients with NIDCM, compared to LGE alone. They examined 85 patients with dilated cardiomyopathy and 107 patients with hypertrophic cardiomyopathy and concluded that galectin-3 is directly involved in cardiac remodeling and progression of heart failure. Additionally, the galectin-3 level together with the LGE status are capable of predicting clinical outcomes in patients with NIDCM [28]. The prognostic value of Gal-3 for long-term events among patients with HF and preserved ejection fraction were confirmed by French et al. They compared the prognostic accuracy of galectin-3, soluble ST2 (sST2) (a member of the superfamily of interleukin (IL)-1 receptors), troponin I and BNP at 1 and 5 years in 1385 patients with reduced, preserved and recovered LVEF. At 5 years, BNP was the most accurate discriminator of risk among patients with reduced and recovered LVEF, while Gal-3 was the most accurate among those with preserved LVEF [29]. In a recent study, Binas et al. investigated the role of galectin-3 and sST2 in patients with non-ischemic dilated cardiomyopathy. They included a number of 262 patients with DCM and, after a mean period of observation of 3.9 years, observed that sST2 was independently predictive of all-cause mortality and cardiac mortality, while galectin-3 was not. Instead, the subgroup analysis revealed that galectin-3 is predictive in the inflammatory DCM, whereas sST2 is predictive in idiopathic and inflammatory DCM, and not in familial DCM [30]. Thus, the prognostic performance of each biomarker may vary between DCM subtypes.

### 2.4. Chemerin

Chemerin is a newly discovered adipokine expressed in liver, lung, pancreas and adipose tissues. Several studies have demonstrated that chemerin is associated with inflammation, obesity, metabolic syndrome, coronary artery disease and, recently, with the presence of dilated cardiomyopathy and chronic heart failure [31,32,33,34]. Zhang and collaborators reported that chemerin concentrations significantly were increased in DCM patients compared with healthy controls. Additionally, they observed that chemerin concentrations were associated with inflammation (indicated by increased concentrations of interleukin (IL)-6, tumor necrosis factor (TNF)-α and CRP) and left ventricular dysfunction, which may explain the poor prognosis of patients with high levels of chemerin [33]. There are only a few studies that evaluated the prognostic value of chemerin in patients with cardiovascular diseases. One of them was published by Zhou and coworkers. They evaluated the prognostic value of serum chemerin in 834 patients with chronic heart failure. After a median follow-up period of 524 days, they found that chemerin was a prognostic marker of major adverse cardiac events in patients with chronic heart failure and *N*-terminal pro-B-type natriuretic peptide values above and below the median [34]. The first study that highlighted the possibility of using chemerin as a prognostic predictor in patients with dilated cardiomyopathy was recently published by Chen et al. The authors included 214 patients with DCM, and after a median follow-up of 18 months, revealed that patients with higher chemerin levels had an increased risk of major cardiovascular events. Thus, serum chemerin may serve as a prognostic indicator in patients with DCM, but its importance was not specifically studied in patients with NIDCM [31].

### 2.5. Tumor Necrosis Factor-α (TNF-α)

Tumor necrosis factor-α is a pro-inflammatory cytokine produced by activated macrophages and monocytes. Different studies have demonstrated the role of TNF-α in the pathophysiology of atherosclerosis, myocardial infarction and chronic heart failure [35,36,37]. Elevated TNF-α levels have also been found in patients with DCM, which indicates that TNF-α may be related to the pathogenesis of dilated cardiomyopathy [36]. Based on all these data, a recent metanalysis, which included nine eligible studies (1338 patients and 1677 controls), evaluated the possible association between TNF-α gene polymorphism (G-308A) and susceptibility to DCM. The main result of this metanalysis is that TNF-α gene polymorphism (G-308A) may have an important role in the pathogenesis and progression of dilated cardiomyopathy, especially in Asian populations [35]. However, to date, there are no studies on the implications of TNF-α in risk stratification of patients with DCM.

## 3. Biomarkers of Myocyte Stress/Stretch

### 3.1. B-Type Natriuretic Peptide (BNP) and N-Terminal-pro Hormone BNP (NT-proBNP)

B-type natriuretic peptide is a biomarker produced mainly by ventricular cardiomyocytes and its level increases in response to increased wall stretching, such as pressure overload and volume expansion. NT-proBNP is the best biomarker in the family of natriuretic peptides to diagnose and monitor heart failure and it can also be used to screen for asymptomatic left ventricular dysfunction, because it is larger and has a longer half-life than BNP, making its measurement easier [1,38]. Thus, these two biomarkers are traditionally used for the screening, diagnosis and prognosis of heart failure. Several studies have found that BNP and NT-proBNP are related to all-cause mortality [5,39]. There are few data on the prognostic value of BNP or NT-proBNP in DCM patients. In a recent study, Chmielewski and coworkers evaluated the role of NT-proBNP and high-sensitivity cardiac troponin T (hs-cTnT) in the detection and risk assessment of clinically stable cardiolaminopathy patients. They included 53 patients from 21 families and, after a median follow-up of 1522 days, observed that an increased hs-cTnT level seems the earliest abnormality emerging in the course of cardiolaminopathies. Additionally, elevated NT-proBNP level > 150 pg/mL may be helpful in arrhythmic risk stratification of cardiolaminopathies [39]. Li and collaborators evaluated the significance and the prognostic utility of NT-proBNP in hospitalized patients with DCM. One of their major findings was that NT pro-BNP independently predicts all-cause mortality in DCM patients. Its level was increased in patients in NYHA classes III and IV, with reduced left ventricular ejection fraction and dilated right ventricular and left atrial diameters [19]. One of the studies that investigated the prognostic utility of right ventricular systolic function in patients with NIDCM and its correlation with plasma NT-proBNP levels was published by Tigen et al. Their main conclusion is that combined use of plasma NT-proBNP levels and tissue Doppler-derived right ventricular systolic functional parameters may be helpful in identifying the very high-risk group in NIDCM and in referring them to cardiac transplant/mechanical support immediately [40].

### 3.2. Soluble ST2

ST2 is a member of the interleukin-1 receptor family, a protein secreted by monocytes in response to mechanical strain [9]. ST2 has two forms: a transmembrane receptor (ST2L) and a soluble receptor (sST2). It is a valuable cardiac biomarker used in the assessment of cardiac remodeling and tissue fibrosis in patients with heart failure, myocardial infarction or acute coronary syndromes. An important advantage of ST2 is that its value does not vary with age, sex, body mass index, renal function or heart failure history, as in the case of BNP, NT-proBNP or hs-troponin [41]. Several studies have demonstrated the prognostic utility of soluble ST2 in patients with acutely decompensated heart failure, high levels of sST2 being associated with an increased risk of heart failure complications, such as arrhythmia, pump failure or death, independent of other cardiac biomarkers [42,43,44,45]. Additionally, the prognostic value of sST2 in patients with mild-to-moderate heart failure with LV systolic dysfunction was investigated by Pascual-Figal et al. Their results suggest that elevated sST2 values tend to predict the risk of sudden cardiac death in patients with heart failure and may also improve prognostic strategies in heart failure management [46]. Regarding the prognostic value of sST2 in patients with NIDCM, the study of Binas and coworkers revealed that sST2 predicts all-cause mortality and cardiac mortality, and could be useful especially in patients with inflammatory myocardial disease and viral persistence [30]. Broch and collaborators evaluated a group of NIDCM with similar baseline characteristics as in the previous study and demonstrated that sST2 levels were more related to hemodynamic stress than to the pathophysiological processes, even if elevated sST2 levels were a predictor for death or heart failure transplantation [47]. The value of ST2 and Galectin-3 in the prognostic stratification of patients with NIDCM was also assessed by Wojciechowska et al. They included 107 stable NIDCM patients, and after a mean follow-up period of 4.8 years, observed a correlation between the serum level of ST2 and all-cause mortality and combined endpoint occurrence (death or heart transplantation and left ventricular assist device implantation) in stable NIDCM patients. This ST2 effect was independent of other well-known prognostic factors, such as NT-proBNP. On the contrary, no predictive role of serum Galectin-3 was found [48]. The results of these studies demonstrate the prognostic value of sST2 in patients with NIDCM. Association of sST2 serum levels with outcomes was also recently demonstrated in pediatric dilated cardiomyopathy. You et al. measured the BNP and sST2 levels in 94 patients with pediatric dilated cardiomyopathy and observed an association of sST2 levels with 6-month and long-term adverse events, sST2 being a promising biomarker that is superior for identifying pediatric patients with DCM at high risk, compared with using BNP alone [49]. Thus, soluble ST2 is currently recognized as an important biomarker for prognosis and monitoring of patients with NIDCM, being included in the 2017 American College of Cardiology/American Heart Association update of heart failure guidelines [5].

## 4. Biomarkers of Myocyte Injury

### 4.1. High-Sensitivity Cardiac Troponin T

Cardiac troponin T and I are sensitive and specific markers of myocardial injury. Additionally, this troponin can predict an adverse outcome in patients with dilated cardiomyopathy. However, their clinical use is limited by the low sensitivity of the conventional commercial assay system [50]. Therefore, a highly sensitive commercial assay of cardiac troponin T and hs-cTnT became available and its prognostic role in patients with NIDCM was evaluated in different studies. The prognostic value of the hs-cTnT level in patients with nonischemic dilated cardiomyopathy was demonstrated for the first time by Kawahara et al. They measured the serum levels of conventional cardiac troponin T, hs-cTnT and BNP in 85 NIDCM patients and observed that an elevated serum value of hs-cTnT is a useful prognostic predictor, independent of left ventricular ejection fraction or BNP. Thus, high serum concentration of hs-cTnT reflects ongoing myocardial damage [51]. The study conducted by Baba and coworkers demonstrated that the hs-cTnT serum level provides a better risk stratification in DCM patients. They performed clinical evaluation, including measurements of troponin in 54 patients with DCM under a clinically stable condition and observed that a higher degree of abnormality in hs-cTnT value is associated with a greater risk of cardiac events. Additionally, an abnormal serum level of hs-cTnT is an independent predictor of adverse outcomes and may be a useful marker for the prediction of left ventricular remodeling or reverse remodeling [52] (Table 1).

### 4.2. Heart-Type Fatty Acid Binding Protein (H-FABP)

H-FABP is a low molecular weight cytosolic protein that is rapidly released into the circulation when the myocardium is injured. Studies have demonstrated that patients with chronic heart failure have increased serum levels of H-FABP, suggesting the presence of ongoing myocardial damage [53,54,55]. Additionally, considering the presence of its high levels in patients with advanced heart failure, H-FABP is considered a novel biomarker for myocyte injury and prognosis in chronic heart failure patients [53]. Regarding the sensitivity and the importance of H-FABP in the detection of ongoing myocardial damage in patients with chronic heart failure, Niizeki et al. demonstrated that H-FABP levels at admission were independently associated with cardiac deaths and nonfatal cardiac events, H-FABP being more sensitive to detect ongoing myocyte injury and to identify patients at high risk compared to TnT [54]. The utility of H-FABP levels to predict critical cardiac events for patients with idiopathic dilated cardiomyopathy was evaluated by Komamura et al. They measured the serum concentrations of H-FABP, brain natriuretic peptide and cardiac troponin T in 92 patients with NIDCM. After a follow-up period of four years, they demonstrated that serum concentration of H-FABP before discharge independently predicted the long-term risk of critical cardiac events in patients with NIDCM, with a comparable predictive power to that of BNP [53]. Moreover, use in clinical practice of H-FABP and BNP combination provides better prognostic value, and their elevated levels suggest a worse prognosis in NIDCM patients [10]. Thus, H-FABP provides insights into ongoing myocardial damage and adverse cardiac remodeling, identifying NIDCM patients at high risk.

### 4.3. Myosin Binding Protein-C (MyBP-C)

Cardiac myosin binding protein-C is a sarcomeric protein with important implications in the regulation of cardiac systolic and diastolic function. Considering that DCM manifests as a deterioration of systolic and diastolic function, more and more studies are being done to better understand MyBP-C implications in patients with dilated cardiomyopathy [56]. Different MyBP-C genetic defects or an eliciting autoimmune response may lead to dilated cardiomyopathy. The autoimmune response causes production of autoantibodies that act on the contractile cardiac protein. The final result will be an autoimmune myocarditis that will ultimately progress to dilated cardiomyopathy and heart failure [57,58]. To our knowledge, to date, there have been no studies on the implications of MyBP-C in risk stratification of patients with NIDCM.

## 5. Biomarkers of Extracellular Matrix Remodeling

### Matrix Metalloproteinases (MMP)

Matrix metalloproteinases are enzymes capable of degrading extracellular matrix proteins. They have an important role in tissue remodeling associated with different physiological or pathological processes as morphogenesis, angiogenesis or tissue repair. Patients with dilated cardiomyopathy have an important myocardial tissue remodeling, which is associated with an extensive reorganization of the cardiac extracellular matrix. One of the first studies that evaluated the cardiac extracellular matrix remodeling by analyzing the matrix metalloproteinases in DCM patients, with a focus on their possible prognostic value, was published by Franz et al. They measured the serum levels of matrix metalloproteinase 9, tissue inhibitor of metalloproteinase 1, fetal tenascin-C and fibronectin, markers known to be involved in the reorganization of the extracellular matrix, and demonstrated that an increase of every single of these parameters is significantly related to a lower survival of DCM patients [59]. In another recent study, Antonov et al. studied the expression of matrix metalloproteinases MMP-1, MMP-9 and their inhibitor TIMP-1 in myocardial autopsy samples, as markers of dilated cardiomyopathy in patients of different ages. They observed an increased expression of these markers by 1.5 to 9.0 times in patients with dilated cardiomyopathy, suggesting that they can be promising predictors of DCM and can be used for the evaluation of the effectiveness of treatment [60].

## 6. Biomarkers of Oxidative Stress

### Myeloperoxidase (MPO)

Myeloperoxidase is a leukocyte-derived enzyme produced by monocytes, neutrophils and endothelial cells [61,62]. It is a marker of oxidative stress, inflammation, endothelial dysfunction and atherosclerosis, being used as a biomarker in different cardiovascular pathologies, such as heart failure or coronary artery disease. MPO levels may predict the presence of heart failure, independent of other markers, such as age and BNP [63,64]. In a study that included patients with LVEF < 35%, MPO was related to poor right ventricular systolic function as well as to the diastolic dysfunction [65]. Additionally, it has been shown that plasma MPO levels are elevated in patients with heart failure and also correlate with disease severity, as reflected by the positive relationship with NYHA functional class. In heart failure patients, MPO has an additive prognostic value over NT-proBNP testing, its principal prognostic value being in patients with intermediate NT-proBNP levels [64,65]. A significant association between MPO levels and H-FABP levels was also observed in patients with chronic heart failure, indicating that increased MPO levels may contribute to ongoing myocardial damage in these patients [61]. Thus, MPO may also have important implications in the risk stratification of patients with dilated cardiomyopathy.

## 7. Neurohormones

Several neurohormones, such as norepinephrine, renin, aldosterone, angiotensin 2 and arginine vasopressin, have been described as biomarkers in patients with heart failure [9]. High circulating levels of norepinephrine, plasma renin activity, aldosterone and endothelin-1 have been found in patients with HF, and norepinephrine and plasma renin activity were found to have independent prognostic value and to significantly increase the predictability of the outcome when added to BNP assay [66,67].

Endothelin-1, a novel endothelium-derived potent vasoconstrictive peptide, has been demonstrated to increase in plasma of patients with dilated cardiomyopathy [68,69]. Moreover, Herrmann and coworkers investigated whether genetic polymorphisms of the endothelin system predict survival in dilated cardiomyopathy patients. They evaluated 125 patients with dilated cardiomyopathy and, for the first time, demonstrated a significant relationship between genetic variations in the endothelin receptor and survival. Their results may have important consequences for the identification of high-risk patients [70] (Table 2).

## 8. Other Biomarkers

### 8.1. Endothelial Progenitor Cells (EPCs)

Cardiac endothelial changes that occur in patients with dilated cardiomyopathy are associated with disease progression and outcome. Therefore, more and more efforts are made to identify biomarkers that have prognostic value, such as endothelial progenitor cells (EPCs). As a response to defective vascularization that has been reported in DCM patients, there is a peripherally increased number of EPCs that contribute to vascular repairs in vivo [71,72,73]. Thus, different studies have reported increased circulating levels of EPCs in DCM patients. Theiss et al. measured different circulating CD34^+^ cell populations in patients with dilated cardiomyopathy and ischemic cardiomyopathy. They found an increased number of circulating cells with stem cell markers in DCM patients, compared to healthy individuals and ischemic heart disease patients. Furthermore, they observed that myocardial homing factors were significantly overexpressed in ischemic versus non-ischemic hearts. This may be explained by an impaired endogenous myocardial regeneration via circulating CD34^+^ cell populations, secondary to a lack of upregulation of important myocardial homing factors [74]. These findings were also confirmed by Roura and coworkers. They observed defective vascularization and impaired vasculogenesis (i.e., de novo vascular organization of mobilized EPCs) and angiogenesis (the formation of new blood vessels from pre-existing mature endothelial cells) in DCM patients [75]. There are also encouraging signals that stem cell therapy, on top of optimal medical management, further improves left ventricular ejection fraction, exercise capacity and long-term prognosis of DCM patients [71,76,77].

### 8.2. Bisphenol A (BPA)

Bisphenol A is a widespread component of polycarbonate plastics and epoxy resins, with implications in several cardiovascular diseases [78]. Unfortunately, the widespread use of products with bisphenol A has increased the risk of its accumulation in a variety of human tissues and body fluid, in people of all ages, including human fetuses and infants [79]. Humans are becoming widely exposed to bisphenol A through skin contact and also through the consumption of contaminated water and food [80]. Several studies have reported potential links between bisphenol A exposure and cancer, diabetes, neuroendocrine, reproductive or cardiovascular diseases [81,82,83,84]. The first study that highlighted the possible association between higher BPA exposure and an increased risk of dilated cardiomyopathy were published by Xiong et al. They measured the serum BPA levels, total testosterone, sex hormone-binding globulin and estradiol levels in 88 DCM patients and 88 age- and gender-matched healthy controls. Their results demonstrated that serum BPA levels are significantly higher in DCM patients than in healthy controls, while total testosterone levels are lower. Additionally, sex hormone-binding globulin (SHBG) had a positive association with BPA [85]. These findings may explain the higher incidence of dilated cardiomyopathy in men compared to women. Thus, considering that androgen action may lower serum SHBG levels while estrogen action increases it, the estrogenic action of BPA may lead to an elevated level of SHBG. The relation between SHBG and cardiovascular disease was also demonstrated by Pascual-Figal et al. They found that elevated SHBG levels were associated with the severity of heart failure and also with a higher risk of cardiac death [86].

### 8.3. MicroRNAs (miRNAs)

Circulating microRNAs are small non-coding RNAs that influence protein translation and has gained much interest as potential genomic biomarkers. Their evaluation could help with the differentiation of the etiology behind heart failure (ischemic vs. nonischemic) and possibly with the prediction of the future course of the disease [87,88]. Although current evidence is lacking, there are some small studies that discovered high plasma levels of miRNA-16 in patients with ischemic and familial dilated cardiomyopathy, confirming its relationship with these etiologies [89,90,91].

### 8.4. Syndecan-1

Cardiac fibrosis is common in patients with NIDCM, being a progressive process, associated with important implications on prognosis and mortality. Syndecan-1 is a member of transmembrane proteoglycan family, involved in cell–matrix interactions and is believed to regulate cardiac fibrosis. Studies have shown that circulating syndecan-1 levels are correlated with markers of fibrosis and remodeling, patients with elevated levels having poor renal function [92,93]. Additionally, a doubling of syndecan-1 levels was associated with an increased risk of all-cause mortality and rehospitalization for heart failure, in patients with CHF with preserved ejection fraction [94]. An interesting study conducted by Liu and coworkers has shown that in patients with heart failure and NIDCM, syndecan-1 level is correlated with fibrosis and inflammatory biomarkers. Moreover, syndecan-1 level had good discriminatory capacity for predicting major adverse cardiac events during a mean follow-up period of 937 days, being an independent risk factor [92].

### 8.5. T-Cadherin

Patients with severe heart failure develop different changes in adipokine levels, progressive catabolism and long-term systemic inflammation. T-cadherin is an adiponectin receptor expressed in the heart and blood vessels [95]. Considering the changes in adipokine levels that appear in patients with chronic heart failure, a recent study aimed to evaluate whether the myocardial T-cadherin levels are associated with the severity of heart failure or might predict outcomes in patients with NIDCM. Their major result was that reduced cardiac T-cadherin levels might be an additional marker of chronic heart failure severity. The role of T-cadherin in the outcome prediction of patients with NIDCM has not been demonstrated in this small number of 29 patients included in the study, but the provided observations might help in future larger clinical studies [96].

### 8.6. Growth Differentiation Factor-15 (GDF-15)

Growth differentiation factor-15 is a stress-responsive cytokine and is emerging as a biomarker implicated in fibrosis, inflammation and ventricular remodeling. Recent studies evaluated the role of GDF-15 as a biomarker of myocardial fibrosis in patients with NIDCM [97], its correlations with worsening functional capacity in idiopathic dilated cardiomyopathy [98] and its role for risk stratification of arrhythmic death in NIDCM [99]. Lok et al. evaluated the GDF-15 levels in patients with NIDCM during left ventricular assist device support and observed that elevated circulating levels of GDF-15 correlate with the degree of myocardial fibrosis and decrease strongly to near-normal levels within 1 month of left ventricular assist device support [97]. In another recent study, Nair and Gongora analyzed the correlations of GDF-15 with worsening functional capacity as well as the interactions between GDF-15 and other markers of fibrosis, in patients with idiopathic dilated cardiomyopathy. Thus, GDF-15 appears to have significant correlations with markers of fibrosis and remodeling, such as soluble ST2 and MMP [98]. An interesting study conducted by Stojkovic and coworkers evaluated the prognostic value of GDF-15 and soluble ST2 for the prediction of fatal arrhythmic events and all-cause mortality in patients with NIDCM. After a median follow-up time of 7 years, they observed that GDF-15 was found to be an independent predictor of fatal arrhythmic events and all-cause mortality. Furthermore, assessment of GDF-15 in addition to left ventricular ejection fraction, could provide supplementary information regarding long-term risk stratification and help identifying patients with NIDCM at risk of arrhythmic death [99].

## 9. Emerging Approaches and Future Directions

Despite recent advances, non-ischemic dilated cardiomyopathy still remains a disease with unacceptable morbidity and mortality, possibly due to the complex pathophysiology. Thus, novel biomarkers addressing specific paths of damage are of urgent need for identification of patients at higher risk of pump failure and sudden cardiac death. In the last years, a growing number of biomarkers have been studied and proposed as potentially useful for risk stratification of patients with dilated cardiomyopathy, but none of them resemble the characteristics of an ideal biomarker. However, existing evidence proves biomarker evaluation to be a very promising tool in risk stratification of patients with dilated cardiomyopathy. We consider that the future holds great promise in improving risk stratification by the incorporation of clinical data, genetic data, imaging data, data from wearable and implantable cardiac devices and serum biomarkers in artificial intelligence algorithms for diagnosis, risk stratification, prognosis and therapeutic management purposes.

## 10. Conclusions

In conclusion, the results presented in this review are of great importance, since it shows that serum biomarkers can be used for risk stratification and for estimating the prognosis of patients with dilated cardiomyopathy. This is essential at the time point of initial clinical presentation because patients with an unfavorable prognosis could be targeted for strict monitoring and more intensive treatment. Assessment of some of these biomarkers might improve arrhythmic risk stratification of patients with NIDCM and appropriate patient selection for prophylactic ICD implantation. The need to find such biomarkers lies in the fact that an optimal heart failure treatment is frequently initiated much too late in patients with non-ischemic dilated cardiomyopathy, despite the use of increasingly advanced diagnostic tools.

## Figures and Tables

**Table 1:** Studies relating well-known biomarkers for risk stratification in dilated cardiomyopathy.

Scheme	Population and Follow-Up	Characteristics	Clinical End Points	Results
**High-sensitivity Cardiac Troponin-T (hs-cTnT)**				
Kawahara et al. [51]	- 85 patients with NIDCM, prospective study, 4.1 years	- 68.2% males, 59.1 ± 15 years; - LVEF 33.9% ± 7.6.	- 23.53% cardiac deaths	- hs-cTnT was elevated (≥0.01 ng/mL) in 54% of the survivors and in 85% of the non-survivors, independent of LVEF or BNP.
Baba et al. [52]	- 54 patients with DCM, prospective study, 5.1 ± 1.6 years	- 79.6% males, 61.2 ± 13.5 years; - LVEF 37.9% ± 11.8.	- 11.11% HF deaths; - 3.70% SCD; - 14.81% HF hospitalizations.	- elevated hs-cTnT values were associated with a greater risk of adverse cardiac events (*p* = 0.003), being useful for the prediction of LV remodeling or reverse remodeling.
***N*-terminal-pro Hormone BNP (NT-proBNP)**				
Tigen et al. [40]	- 75 NIDCM patients, prospective study, 29 ± 16 months	- 69.3% males, 40 ± 15 years; - LVEF 25.4% ± 7.4.	- 38.66% HF deaths; - 8% SCD; - 6.66% heart transplantation.	- 92.5% patients with clinical end points had right ventricular systolic dysfunction and/or plasma NT-proBNP > 1700 pg/mL; both of these parameters may help identify the very high-risk NIDCM patients.
Li et al. [19]	- 622 DCM patients, retrospective cohort study, 2.6 ± 1.6 years	- 73.5% males, 51.4 ± 14.6 years; - LVEF 31.1% ± 8.4.	- 21.1% all-cause mortality.	- plasma NT pro-BNP and hs-CRP were strong predictors of all-cause mortality in DCM patients, independent of age, LV diameter, NYHA functional class and LVEF.
**Soluble ST2 (sST2)**				
Binas et al. [30]	- 262 DCM patients (44.7%-iDCM, 33.2%- ivDCM, 22.1%-fDCM) prospective cohort study, 3.9 years	- 75.2% males, 50.2 ± 12.8 years; - LVEF 30% ± 8.4.	- all cause mortality: 14.5% in iDCM, 17.2% in ivDCM and 19% in fDCM; - cardiac mortality: 12% in iDCM, 11.5% in ivDCM and 15.5% in fDCM.	- sST2 predicts all-cause mortality and cardiac - mortality in patients with NIDCM and could be - useful especially in patients with inflammatory - myocardial disease and viral persistence.
Broch et al. [47]	- 102 NIDCM patients, prospective study, 3.5 years	- 73% males, 51 ± 14 years; - LVEF 26% ± 10.	- 2.1% HF deaths; - 2.1% SCD; - 6.31% heart transplantation.	- sST2 was elevated in patients with severe symptoms, but did not vary with etiology, viral presence or the amount of myocardial fibrosis; thus, sST2 reflects hemodynamic stress rather than pathogenic processes in the myocardium.
Wojciechowska et al. [48]	- 107 NIDCM patients, prospective study, 4.8± 0.4 years	- 80.37% males, 50.3 ± 17.5 years; - LVEF 25% ± 10.	- 25.2% deaths; - 11.2% heart transplantation; - 0.9% LVAD.	- increased sST2 levels are associated with death, heart transplantation and LVAD implantation, independent of NT-proBNP.
**Galectin-3 (Gal-3)**				
Vergano et al. [25]	- 150 NIDCM patients, prospective cohort study	- 73% males, 58 ± 14 years; - LVEF 35% ± 13.	- not evaluated	- Gal-3 is involved in cardiac fibrosis and remodeling, contributing to the progression of the HF and in the selection of high-risk NIDCM patients.
Hu et al. [28]	- 192 NIDCM patients (85 with DCM and 107 with HCM), prospective study, 7 years.	- 75.5% males, - 53.77 ± 15.07 years; - LVEF 42.6% ± 13.5 in DCM patients.	- in DCM patients: 4.7% deaths, 18.82% arrhythmic events and 7.05% aggravated HF.	- galectin-3 is involved in cardiac remodeling and progression of HF; Gal-3 with LGE status is capable of predicting clinical outcomes in patients with NIDCM

DCM—dilated cardiomyopathy; iDCM—idiopathic dilated cardiomyopathy; ivDCM—inflammatory and/or viral dilated cardiomyopathy; fDCM—familial dilated cardiomyopathy; HCM—hypertrophic cardiomyopathy; HF—heart failure; LV—left ventricle; LVAD—left ventricular assist device; LVEF—left ventricular ejection fraction; NIDCM—non-ischemic dilated cardiomyopathy; NT pro-BNP—*N*-terminal-pro hormone brain natriuretic peptide; NYHA—New York Heart Association; SCD—sudden cardiac death.

**Table 2:** Studies related to novel possible biomarkers for risk stratification in dilated cardiomyopathy.

Scheme	Population and Follow-Up	Characteristics	Clinical End Points	Results	Limitations
**High-sensitivity-CRP (hs-CRP)**					
Li et al. [19]	- 622 patients with DCM, retrospective cohort study, 2.6 ± 1.6 years.	- 73.5% males, 51.4 ± 14.6 years; - LVEF 31.2% ± 8.5.	- 33.6% all-cause mortality.	- plasma NT pro-BNP and hs-CRP at admission were strong predictors of all-cause mortality in DCM patients, independent of age, LV diameter, NYHA functional class and LVEF.	- biomarkers were measured only at admission.
Chitose et al. [16]	- 84 patients with NIDCM, prospective study, 42 months.	- 80.95% males, 55.9 ± 1.5 years; - LVEF 33.8% ± 1.0.	- 27.38% had cardiac events; - 21.42% died of cardiac causes.	- hsCRP is a useful prognostic marker in NIDCM patients, independent of hemodynamics and BNP.	- hsCRP was evaluated using only one point sampling and was not observed after treatment for HF.
**Neutrophil/Lymphocyte ratio (NLR)**					
Avci et al. [22]	- 87 patients with idiopathic DCM, prospective study.	- 62.06% males, 48.7 ± 13.8 years; - LVEF 29.7% ± 6.9.	- not evaluated.	- NLR was significantly higher in patients with NYHA III or IV.	- small sample size; - other inflammatory markers were not measured.
Araujo et al. [12]	- 57 pediatric patients with DCM, retrospective study, follow-up 46 ± 14 months.	- 40% males, 48 ± 55.9 months, higher incidence in infants < 12 months; - LVEF 35.5% ± 9.8.	- 28.1% died or were submitted to cardiac transplant.	- high NLR was associated with poor prognosis and a higher risk of death or submission to cardiac transplant.	- small sample size; - absence of a control group; - a single sample of each patient.
**Chemerin**					
Chen et al. [31]	- 214 patients with DCM, prospective study, 18 months	- 61.2% males, 55 ± 7 years; - LVEF 36% ± 6.	- MACEs (cardiac mortality, stroke and myocardial infarction): 30.8% - all cause mortality: 16.8%.	- higher serum chemerin was associated with an increased risk of MACEs.	- a single measurement of serum chemerin; - NYHA functional class was not collected.
**Tumor Necrosis Factor-α (TNF-α)**					
Chen et al. [35]	- 1338 patients and 1677 controls from 9 studies, systematic metanalysis.	- 67.58% males, 52.78 years.	- not evaluated.	- TNF-α G-308A polymorphism may play an important role in the pathogenesis and progression of DCM, especially in Asian populations.	- small or moderate sample sizes of the studies; - 3 studies included ischemic, valvular or viral DCM.
**Heart-type Fatty Acid Binding Protein (H-FABP)**					
Komamura et al. [53]	- 92 patients with NIDCM, prospective study, 48 months	- 71% males, 48.5 years; - LVEF 30% in non-survivors and 37% in survivors (*p* = 0.002).	- cardiac death: 14.13%; - heart transplant: 7.6%; - left ventricular assist device: 3.26%.	- H-FABP before discharge independently predicted the long-term risk of critical cardiac events in NIDCM	- small sample size.
**Matrix Metalloproteinases (MMP)**					
Franz et al. [59]	- 187 patients with DCM, prospective study, 3–6 months	- 78.07 % males, 48.9 ± 12.2 years; - LVEF 31.7% ± 12.4.	- death or cardiac transplantation was higher in patients with elevated MMP-9 serum values.	- increased serum levels of MMP 9, tissue inhibitor of MMP 1, fetal tenascin-C and fibronectin are related to a lower survival of DCM patients	- small sample size.
**Endothelin-A (ETA)**					
Herrmann et al. [70]	- 125 patients with DCM, prospective study, 40 ± 21 months	- 79.2 % males, 58 ± 10 years; - LVEF 32% ± 12.	- death was higher in carriers of ETA T alleles.	- role of a genetic variation in the ETA receptor on survival in DCM patients.	- small sample size.

DCM—dilated cardiomyopathy; HF—heart failure; LV—left ventricle; LVEF—left ventricular ejection fraction; MACE—major adverse cardiovascular events; NIDCM—non-ischemic dilated cardiomyopathy; NT pro-BNP—*N*-terminal-pro hormone BNP; NYHA—New York Heart Association.
